# Association between the site of clear corneal Phakic intraocular lens implantation incisions and the inflow of ocular surface fluid into the anterior chamber

**DOI:** 10.3389/fmed.2023.1063003

**Published:** 2023-02-27

**Authors:** Huan Wan, Yunhan Tao, Jianan Duan, Lin Wang, Li Tang

**Affiliations:** ^1^Department of Ophthalmology, West China Hospital of Sichuan University, Chengdu, Sichuan, China; ^2^Department of Ophthalmology, People's Hospital of Meishan, Meishan, Sichuan, China

**Keywords:** myopia, phakic intraocular lens implantation, clear corneal incision, anterior chamber, intraocular inflammation, endophthalmitis

## Abstract

**Background:**

Posterior chamber phakic intraocular implantable collamer lens (ICL) implantation is an effective surgical option for the management of myopia. Over the past few years, the number of ICL surgeries has significantly increased. Postoperative inflammation and endophthalmitis are among the most serious complications after successful ICL surgery. Sometimes, when the blepharospasm is removed at the end of surgery, a small amount of the ocular surface fluid will flow into the anterior chamber, which can lead to an increased risk of infection and inflammation. However, little attention has been paid to this phenomenon.

**Purpose:**

We conducted a retrospective study to compare the incidence of extraocular fluid inflow into the eye through the clear corneal incision (CCI) at two different positions (superior and temporal sides).

**Methods:**

A total of 116 patients with myopia underwent superior CCI ICL implantation (*n* = 58) or temporal CCI ICL implantation (*n* = 58) at our hospital from October to December 2021. The incidence of conjunctival sac fluid entering the anterior chamber after eyelid fixative was removed was compared in both groups.

**Results:**

Both groups were well matched in all parameters. Ocular surface fluid inflow into the anterior chamber was significantly higher in the superior CCI group (25/58, 47.1%) than in the temporal CCI group (1/58, 1.7%) (*p* < 0.05).

**Conclusion:**

In the patients with ICL implantation, the temporal CCI was better than the superior CCI in avoiding the ocular surface fluid inflow into the anterior chamber, and the potential risk of infection and inflammation was lower.

## 1. Introduction

Posterior chamber phakic intraocular lens implantation (EVO Visian implantable collamer lens (ICL) V4c, STAAR Surgical, Monrovia, CA, USA) in phakic eyes is widely considered a safe and effective refractive surgery for the correction of moderate-to-high ametropia (1–3). However, there are still some complications of this operation that should be avoided. Postoperative inflammation and endophthalmitis are among the most serious complications that usually lead to poor visual outcomes. Although endophthalmitis after ICL surgery is rare, with an incidence of 1/6,000 (4), it can lead to blindness. Several factors are thought to contribute to the incidence of endophthalmitis following ICL, including intraoperative surgical complications, poor wound construction, contamination of the conjunctiva, implant contamination, and hypoimmunity (5). In particular, the incision location is an important factor that may increase the risk of endophthalmitis (6, 7). Because of the different facial features of patients of different races, temporal clear corneal incision (CCI) is generally preferred by European and American ophthalmologists, while superior CCI is preferred by some Asian ophthalmologists. Furthermore, we found an interesting phenomenon; on occasion, a small amount of ocular surface fluid can backflow into the anterior chamber when the operator removes the blepharoplasty at the end of the surgery, which may lead to postoperative inflammation or infection. However, this phenomenon has generally been ignored, and to date, it has not been reported in detail.

We hypothesized that the difference in the location of the CCI may be an important factor in conjunctival sac fluid inflow. In this study, we aimed to determine whether temporal CCIs have significantly better wound sealing ability than superior CCIs by comparing the occurrence of conjunctival sac fluid entering the anterior chamber when the eyelid fixing piece is removed at the end of the operation.

## 2. Methods

### 2.1. Study design and patients

Between October and December 2021, a total of 116 patients with myopia who underwent ICL implantation at our hospital were included in the study. The retrospective clinical study was approved by our hospital’s Biomedical Ethics Review Committee and adhered to the principles of the Helsinki Declaration. All participants were between the ages of 18 and 40 years and had an anterior chamber depth > 2.8 mm, anterior chamber angle 30° to 40°, and corneal diameter > 10.6 mm. Patients with keratopathy, history of previous eye surgery (including eyelid surgery), eyelid abnormality, and pupil displacement were excluded. Patients were divided into two groups based on the position of CCI. A total of 58 patients underwent temporal CCI and 58 underwent superior CCI.

### 2.2. Treatment

A complete ophthalmologic examination was performed the day before surgery. Preoperative assessments included best corrected visual acuity, intraocular pressure, slit lamp examination, fundus examination, ultrasound biomicroscopy, corneal topography, anterior segment optical coherence tomography, and B-scan ultrasonography. Topical antibiotic drops were administered prior to surgery and were continued for 1 week postoperatively. All surgeries were performed by the same surgeon with experience in phakic intraocular lens implantation. The surgeon was right-hand dominant. On the day of surgery, the patient underwent pupil dilation before surgery. After topical anesthesia by the Promecaine hydrochloride eye fluid, a standard two-plane 2.8-mm CCI(2.8 mm 45°Bevel Up(BVI Beaver) Xstar Safety Slit Knife) was created, and the incision tunnel was 2 to 3 mm long. Except for the incision site, all other surgical steps in the two groups were identified as follows: ICL was inserted into the anterior chamber, viscoelastic material (iviz MEDICAL SODIUM HYALURONATE GEL) was injected into the anterior chamber, and the ICL was adjusted to the posterior chamber. The viscoelastic agent was then completely replaced with a balanced salt solution. A watertight corneal incision was left unsutured. At the end of the operation, two ophthalmologists evaluated the self-sealing properties of CCI. When the lid retractor was removed, the same two ophthalmologists observed and recorded whether the ocular surface fluid flowed into the anterior chamber again under the microscope (Zeiss Lumera 700). All operations were videotaped for review. Post-operative follow-up was administered regularly, and any changes in ocular inflammation and visual acuity were recorded.

The association between surgical incision location and the incidence of ocular surface fluid inflow into the anterior chamber was determined using the chi-square (χ^2^) test. The statistical significance was evaluated at 0.05 level, and all analyses were performed using SPSS 26.0 (SPSS Inc., Chicago, IL, United States).

## 3. Results

**Figure 1:**
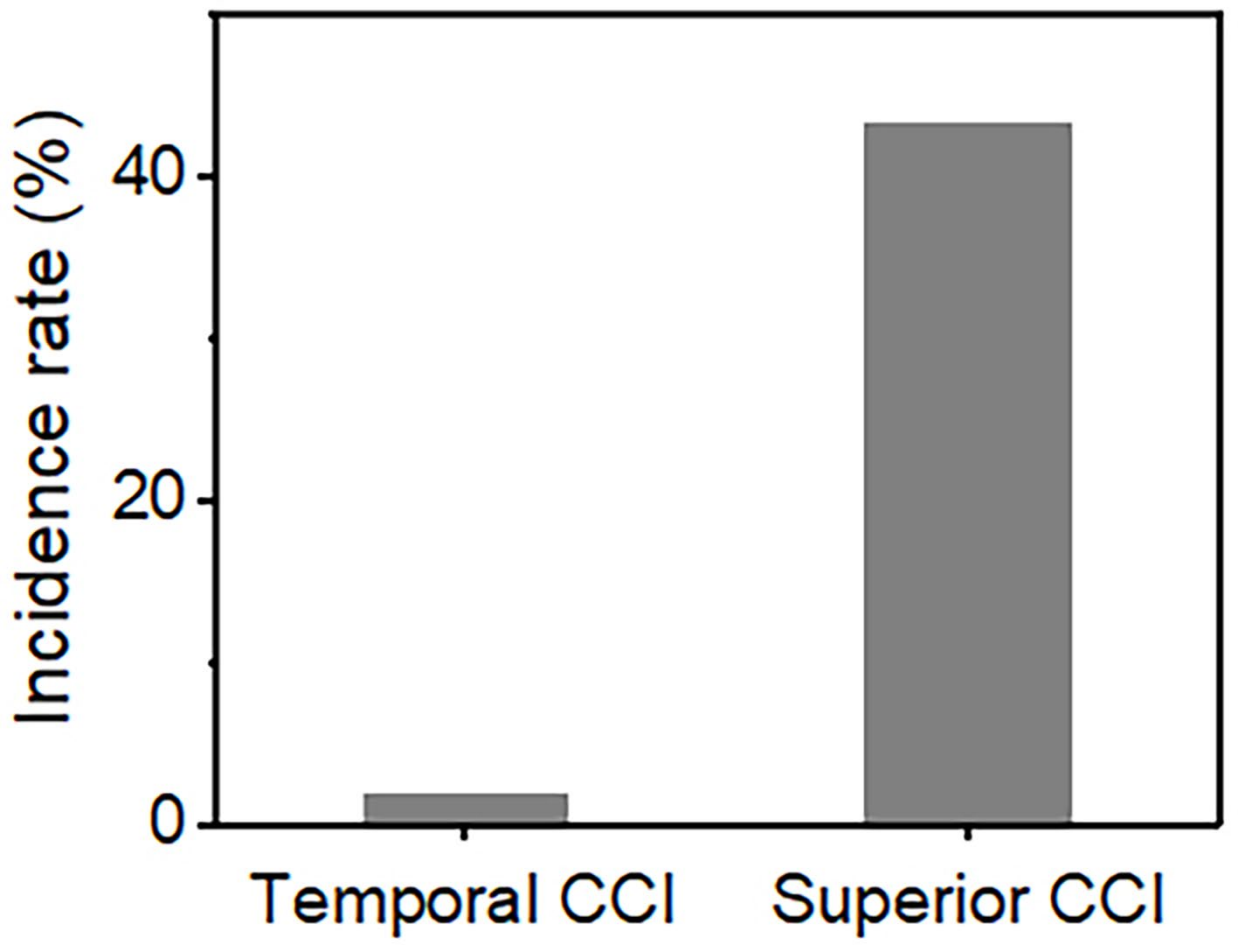
The incidence of postoperative conjunctival sac fluid inflow into the anterior chamber.

**Figure 2:**
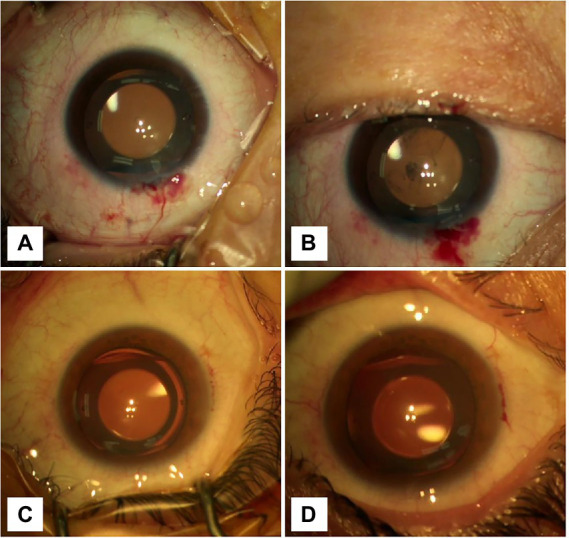
Graphs showing the changes in the anterior chamber before and after removing the blepharostat. **(A)** The CCI was located at 12 o’clock. The anterior chamber was clean, and the incision was well self-sealed before blepharospasm removal. **(B)** The CCI was located at 12 o’clock. After removing the blepharospasm, the conjunctival sac hemorrhagic fluid flowed into the anterior chamber. **(C)** The CCI was located at 9 o’clock. The anterior chamber was clean, and the incision was well self-sealed before blepharospasm removal. **(D)** The CCI was located at 9 o’clock. After removing the blepharospasm, any visible inflow of orbital surface fluid was not seen.

Our study population consisted of 116 patients of whom 58 eyes underwent temporal incision ICL and 58 eyes underwent superior incision ICL. Baseline demographic data (age, sex, ratio of right and left eyes, etc.) were similar between the groups (Table 1). In the temporal incision group, there were 15 men and 43 women with an average age of 27.64 years. In the superior incision group, there were 9 men and 49 women with an average age of 28.21 years. No significant difference was noted in the preoperative ocular biometric parameters between the groups, such as anterior chamber depth (*p* = 0.67), white-to-white distance (*p* = 0.506), central cornea thickness (*p* = 0.279), and keratometrics (*p* = 0.52). Conjunctival sac fluid flowing into the anterior chamber was observed in one of 58 (1.7%) in the horizontal CCI group and in 25 of 58 (47.1%) in the superior CCI group (Table 2; Figure 1). Figure 2 and Supplementary Videos 1, 2 show the conjunctival sac fluid inflow into the anterior chamber before and after the removal of the blepharostat, using superior and temporal CCI, respectively. The incidence of conjunctival sac inflow into the anterior chamber was significantly higher in the superior CCI group than in the temporal CCI group (*p* < 0.05).

**Table 1:** Baseline characteristics of patients.

	Temporal CCI	Superior CCI	*p*-value
Number	58	58	–
Sex: Male (*n*, %)	15, 25.86%	9, 15.52%	0.343
Age (mean ± SD) (years)	27.64 ± 5.62	28.21 ± 6.03	0.692
Anterior chamber depth (mean ± SD) (mm)	3.27 ± 0.25	3.27 ± 0.27	0.67
White-to-white distance (mean ± SD) (mm)	11.55 ± 0.36	11.55 ± 0.34	0.506
Central cornea thickness (mean ± SD) (μm)	526.97 ± 34.90	520.83 ± 32.42	0.279
Keratometric (mean ± SD)	44.23 ± 1.61	44.15 ± 1.49	0.52

**Table 2:** Inflow of conjunctival sac fluid into the anterior chamber while removing the blepharoplast at the end of surgery.

	Temporal CCI (*n* = 58)	Superior CCI (*n* = 58)
Negative (−)	57	33
Positive (+)	1	25
Incidence rate (%)	1.7	43.1
Chi-square value	28.554	
*p*-value	0.0000	

SD, Standard deviation, CCI, Clear corneal incision.

## 4. Discussion

Our study showed that ocular surface fluid inflow in the anterior chamber after removing the lid retractors was more common with superior CCI than temporal CCI. Therefore, the self-sealing properties of unsutured temporal CCI were better than superior CCI. This is thus far the first study to report the differences between superior and temporal CCI with respect to the conjunctival sac fluid inflow the anterior chamber. Melvin et al. (8) demonstrated, using laboratory models, that fluid containing ink outside the eye could enter the eye through an unsutured clear corneal incision when intraocular pressure changes or mechanical pressure changes occurred outside the eye (8). In our study, we focused on ICL implantation because of its short operation time, it being a simple procedure with few intraoperative complications, and that the corneal condition is easy to observe. The site of incision used during ICL implantation depends on the patient’s age, total diopter, corneal astigmatism, and other conditions. Surgical incisions are a risk factor for developing intraocular infection. In severe cases, intraocular infection may lead to serious blindness or even enucleation. At the same time, the potential for the development of endophthalmitis is a mental burden for the surgeon. Chung et al. (9) reported a case of endophthalmitis occurring 3 days after posterior chamber toric phakic intraocular lens implantation using a superior CCI; bacterial culture revealed streptococcal infection. Jalili et al. (10) reported a case of aspergillus infection after ICL implantation with the surgical incision at the 12 o’clock (straight superior). Kaur et al. (11) presented a case of endophthalmitis with acute methicillin-resistant staphylococcal infection after ICL implantation; the corneal incision was sutured with one stitch during a second operation. Couto et al. (12) reported a case of endophthalmitis after posterior chamber toric phakic intraocular lens implantation, in which nodules were found at both the primary and lateral incisions. Ultrasound biomicroscopy showed an intraocular granuloma above the surgical incision, suggesting the source of intraocular infection (13). These studies indicate that improper incisions may increase the probability of postoperative intraocular infection.

Resident bacteria flora can be detected in conjunctival sacs of healthy people. Infections may also be caused by opportunistic pathogens present in the conjunctival sac, such as *Staphylococcus epidermidis*, *Staphylococcus aureus*, and various *Streptococcus* spp. (13). Opportunistic infections may occur when immune resistance decreases during eye surgery. We found that the fluid in the conjunctival sac was more likely to flow into the anterior chamber when the blepharospasm was removed after a straight superior CCI at the end of the operation, possibly contaminating the aqueous humor and predisposing it to infection. A superior CCI is more likely to cause bacterial colonization due to eyelid occlusion; this is less likely with a temporal CCI as it allows frequent blinking. In addition, there are many panni at the superior cornea limbus, and the conjunctival sac fluid containing blood cells is prone to flow into the anterior chamber through the superior CCI, which may increase postoperative immune inflammatory reaction. Therefore, further attention should be given in this regard.

There are many reasons for the increased possibility of conjunctival fluid entering the anterior chamber after ICL implantation, including pressure changes (8), degree of astigmatism, eyelid occlusion, and steep angles of surgery (14). Removal of the lid retractors may exert pressure on the posterior wound edge of the superior CCI and cause outflow of aqueous humor; however, it has little influence in temporal CCI. In addition, a low intraocular pressure allows inflow of extraocular fluid into the anterior chamber through the incision tunnel. Similar events may also occur after manual pressure in the early postoperative period such as during rubbing of the eye and opening the eyelids to apply eye drops. Furthermore, occlusion of the forehead when conducting superior CCI may stretch the corneal incision and facilitate conjunctival sac fluid displacement (14).

Superior CCI is favored by some Asian doctors because left and right eye operations can conveniently be performed without moving the position of the microscope and operator. It is also preferred by some inexperienced surgeons as the patient’s forehead provides support to the surgeon’s hands (15). However, with the increased risk of conjunctival sac fluid flow into the anterior chamber, it is important for surgeons to adapt to various incision sites. At present, we recommend non-toric IOL implantation in eyes with astigmatism of less than 1.0 D (16). Thus, the superior CCI is often selected to reduce preoperative corneal astigmatism (15, 17). If superior CCI is chosen, then the incision tunnel can be appropriately extended to prevent conjunctival sac fluid from flow into the anterior chamber through the incision. Harvey et al. (18) demonstrated that femtosecond laser-created CCIs had significantly better wound sealing ability than those created with a metal keratome and was recommended for superior CCI; however, this comes at a higher cost. Our study shows that in the patients with ICL implantation, temporal incision was better than superior incision in avoiding the conjunctival sac water inflow into the anterior chamber, and as a result, the potential risk of infection was lower.

There are, however, limitations to this study. This study only evaluated cases in which the blepharoplasty was removed at the end of surgery. Furthermore, it was a single-center retrospective study. Owing to the low postoperative infection rate of ICL, more study samples and long-term postoperative follow-ups are needed to confirm our findings.

## Data availability statement

The original contributions presented in the study are included in the article/Supplementary material, further inquiries can be directed to the corresponding authors.

## Ethics statement

The studies involving human participants were reviewed and approved by Ethics Committee on Biomedical Research, West China Hospital of Sichuan University. The patients/participants provided their written informed consent to participate in this study. Written informed consent was obtained from the individual(s) for the publication of any potentially identifiable images or data included in this article.

## Author contributions

HW, YT, JD, LT, and LW contributed to the manuscript. All authors contributed to the article and approved the submitted version.

## Conflict of interest

The authors declare that the research was conducted in the absence of any commercial or financial relationships that could be construed as a potential conflict of interest.

## Publisher’s note

All claims expressed in this article are solely those of the authors and do not necessarily represent those of their affiliated organizations, or those of the publisher, the editors and the reviewers. Any product that may be evaluated in this article, or claim that may be made by its manufacturer, is not guaranteed or endorsed by the publisher.

## Supplementary material

SUPPLEMENTARY VIDEO 1 The inflow of extraocular fluid with a superior CCI.

SUPPLEMENTARY VIDEO 2 No inflow of ocular surface fluid was visible with the temporal CCI.
