# Supplementary figures and images for: Association between the site of clear corneal Phakic intraocular lens implantation incisions and the inflow of ocular surface fluid into the anterior chamber
Source: Front Med (Lausanne). 2023 Feb 27;10:1063003. doi: 10.3389/fmed.2023.1063003 (PMC10019354; doi:10.3389/fmed.2023.1063003)

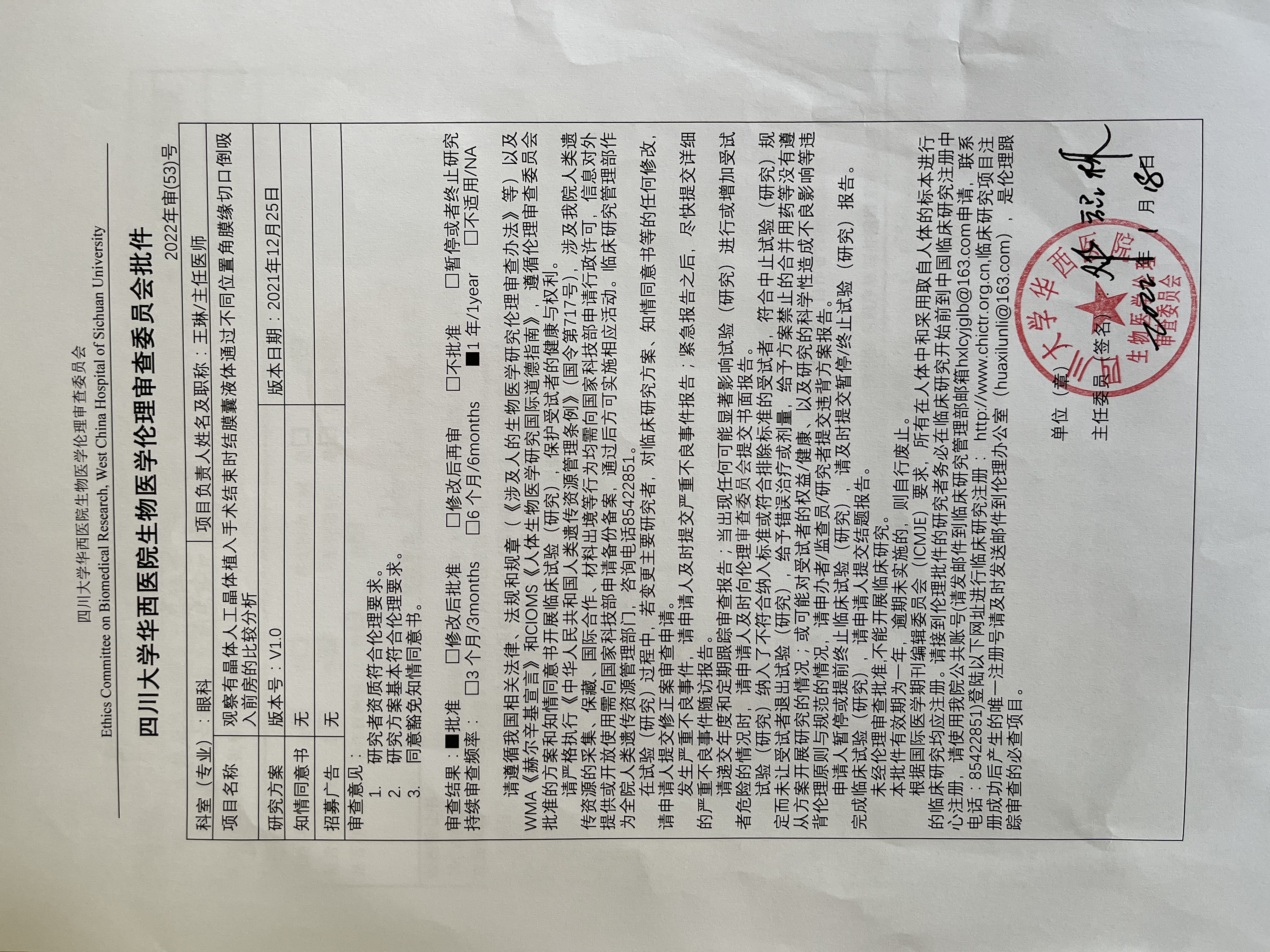

Supplement: Supplementary file 3 [file Image_1.JPEG]
